# Supplementary material for: Identification of Brain-Specific Treatment Effects in NPC1 Disease by Focusing on Cellular and Molecular Changes of Sphingosine-1-Phosphate Metabolism
Source: Int J Mol Sci. 2020 Jun 24;21(12):4502. doi: 10.3390/ijms21124502 (PMC7352403; doi:10.3390/ijms21124502)
Supplement: Supplementary file 1 [file ijms-21-04502-s001.zip › ijms-847768-supplementary-final.docx]

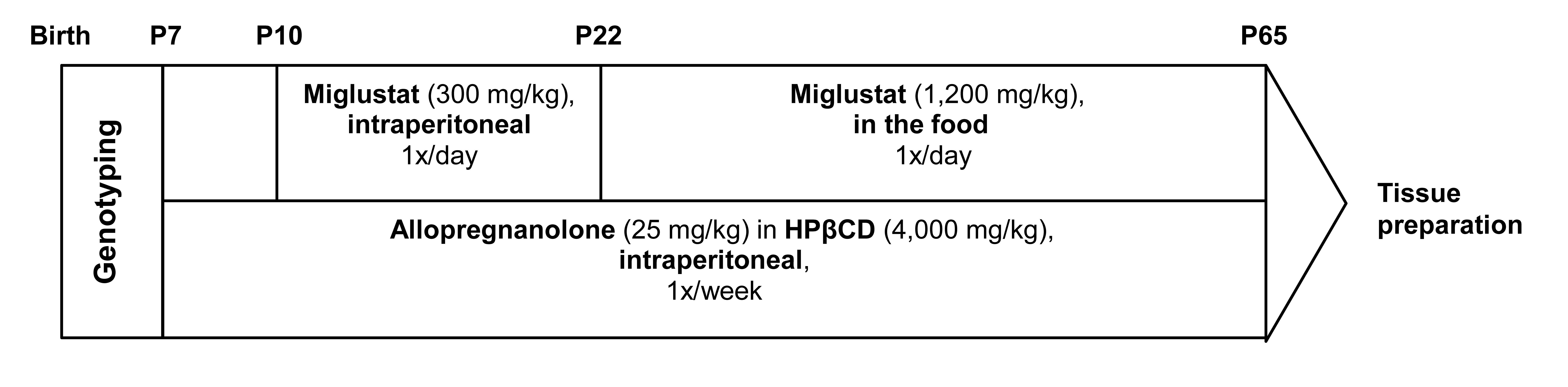


**Figure S1:** Treatment scheme of *Npc1^+/+^* and *Npc1^-/-^* mice, starting at P7 until death at P65. Sham-treated mice received Ringer ‘s solution or 0.9 % normal saline solution. HPßCD: 2-hydroxypropyl-β-cyclodextrin, P: postnatal day


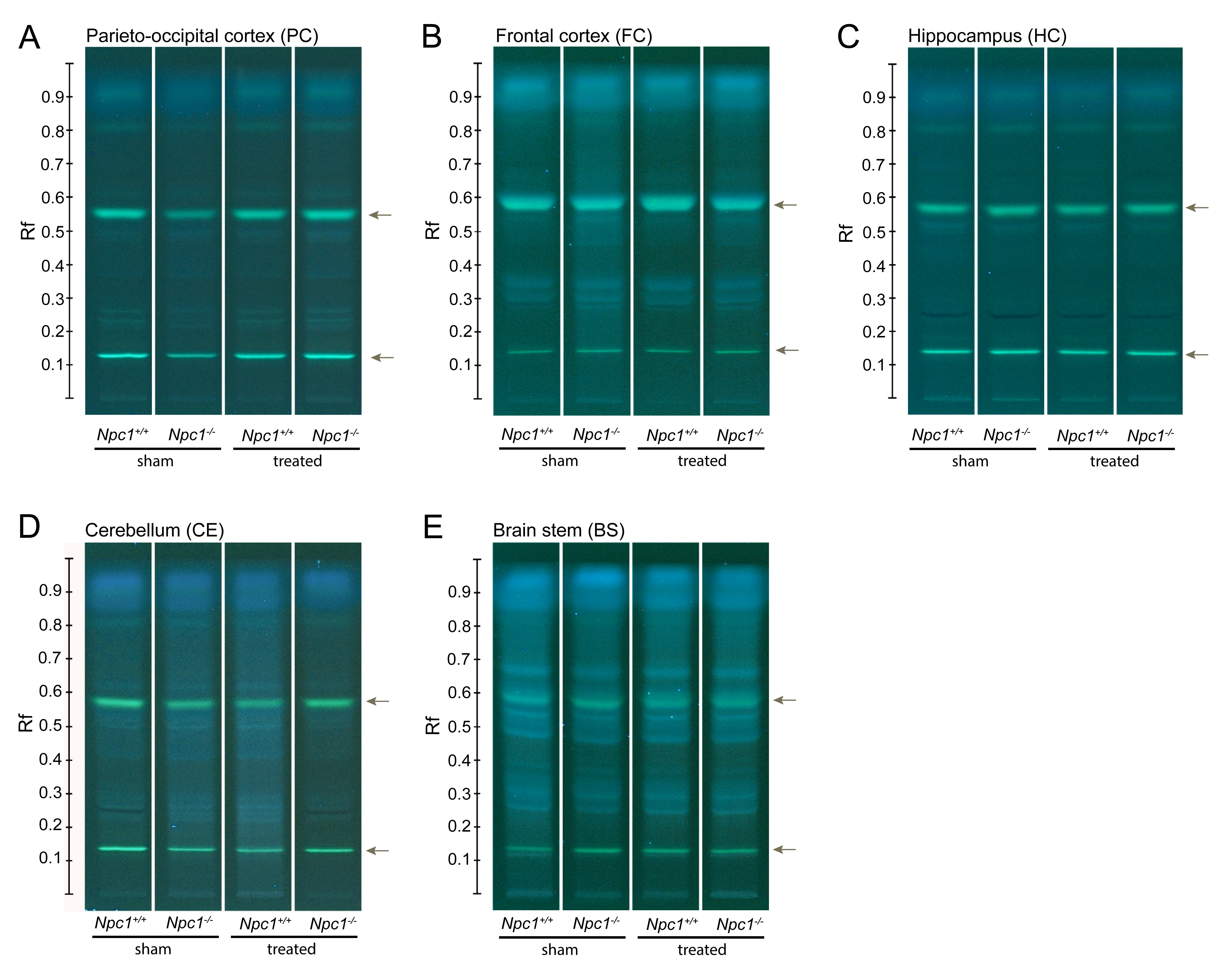


**Figure S2****:** HPTLC (High-performance thin-layer-chromatography) analysis showing Topfluor LPA as internal standard from sham-treated and treated *Npc1^+/+^* and *Npc1^-/-^* mice and different brain regions (**A-E**, all groups *n* = 3). 5 µL/50 mg brain tissue of fluorescent standard Topfluor LPA was added to each sample. Fluorescence was detected with the CAMAG Visualizer at 366 nm. Topfluor LPA bound to the silica gel plate at Rf 0.13 and 0.57. Rf: retention factor LPA: lysophosphatidic acid.





**Figure S3:** Quality of cloned pEGFP-*mS1pr1-5* constructs was proven by gel electrophoresis after enzymatic digestion with the respective restriction enzyme (**A**). Cleavage results in the following fragments. *mS1pr1*: 1154 bp, *mS1pr2*: 1080 bp, *mS1pr3*: 1142 bp, *mS1pr4*: 1168 bp, *mS1pr5*: 1208 bp and pEGFP-N1: 4,7 kb. Uncleaved plasmids (pEGFP-*mS1pr1-5*: 5,8-5,9 kb) were used for control purposes. HEK293H cells were transfected to check the protein expression of S1PR1-5 (**B**). Overexpression of GFP-coupled S1PR1-5 proteins shown via immunocytochemistry confirmed the expression of plasmids. The scale bar represents 10 µm. Western Blot analyses were performed to verify S1PR3 (**C**) and S1PR5 antibody specificity (**D**). Protein lysates of GFP-coupled *mS1pr1-5* transfected HEK293H cells were generated and purified via µMacs columns. Purified lysates were applied and verified with anti-GFP antibody, detecting respective posttranslational modified forms of all GFP-coupled S1PR1-5 proteins. The anti-S1PR3 and anti-S1PR5 antibody were used to detect exclusively their respective protein. No cross-reactivity to other members of the S1PR protein family was detected. Blocking peptide was used to identify specific S1PR3 bands (marked with black boxes, C). The 42 kDa band of S1PR3 was less strongly expressed while using the blocking peptide. Kidney protein lysate was used as control. S1PR3 antibody binding to S1PR3-EGFP protein was blocked by the peptide.


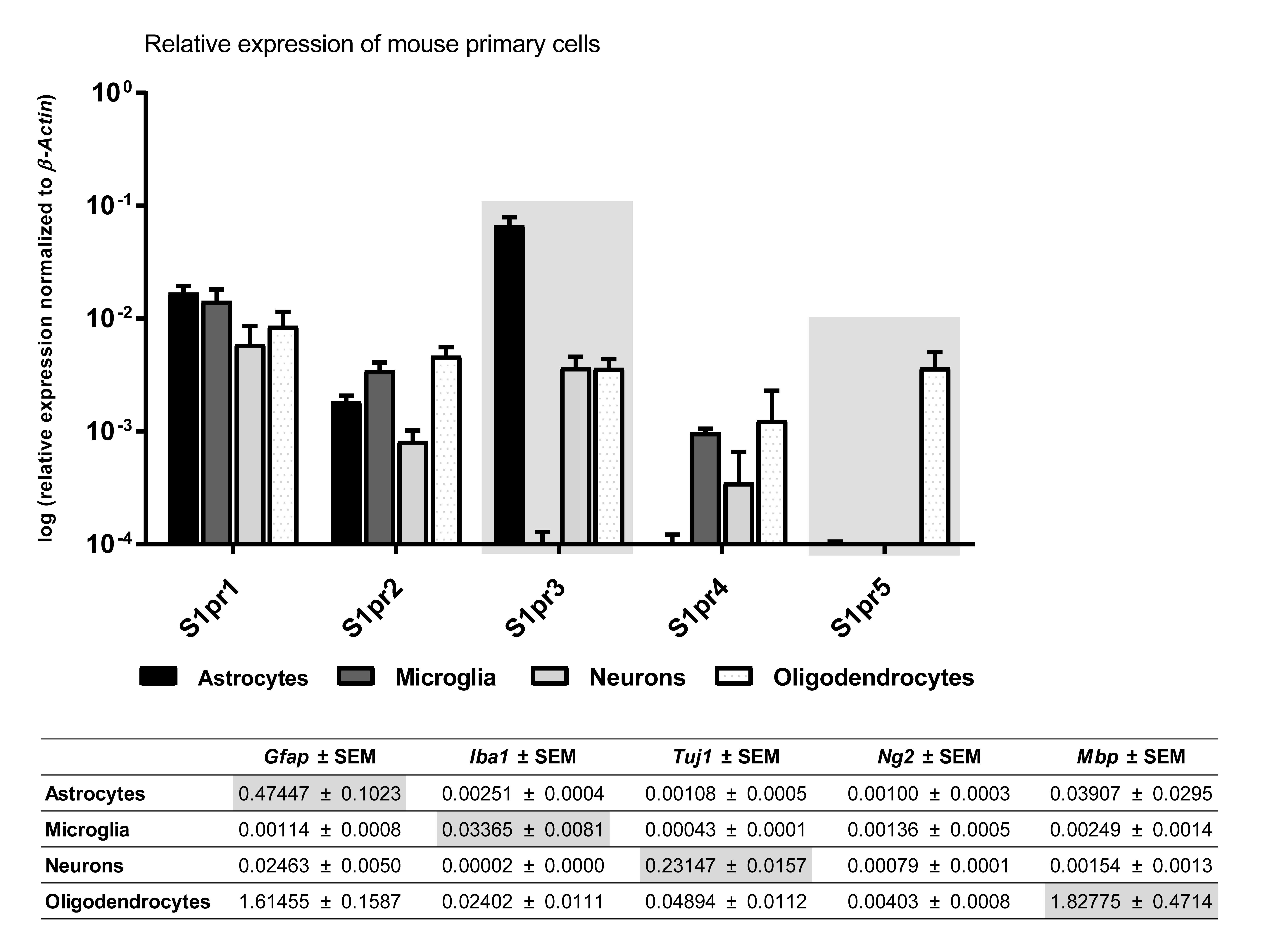


**Figure S4:** Cell-type-specific localization of *S1pr1-5* mRNA in mouse primary cells via qRT-PCR (A). *S1pr1* and *S1pr2* mRNA was expressed in all cell types *S1pr3* mRNA was expressed in neurons, oligodendrocytes, and, most of all, in astrocytes. *S1pr4* mRNA was clearly expressed in microglia, neurons, and oligodendrocytes. *S1pr5* mRNA was expressed exclusively in oligodendrocytes. To verify the purity of mouse primary cells, the expression of specific marker genes was analysed via qRT-PCR: *Gfap* (glial fibrillary acidic protein) for astrocytes, *Iba1* (ionized calcium-binding adapter molecule-1) for microglia, *Tuj1* (β3-tubulin) for neurons, *Ng2* (chondroitin sulfate proteoglycan) for oligodendrocyte progenitors und *Mbp* (myelin basic protein) for mature oligodendrocytes. Data are normalized to *β-Actin* and represented as mean ± SEM. Astrocytes, microglia, neurons (n=3), oligodendrocytes (n=3-8).


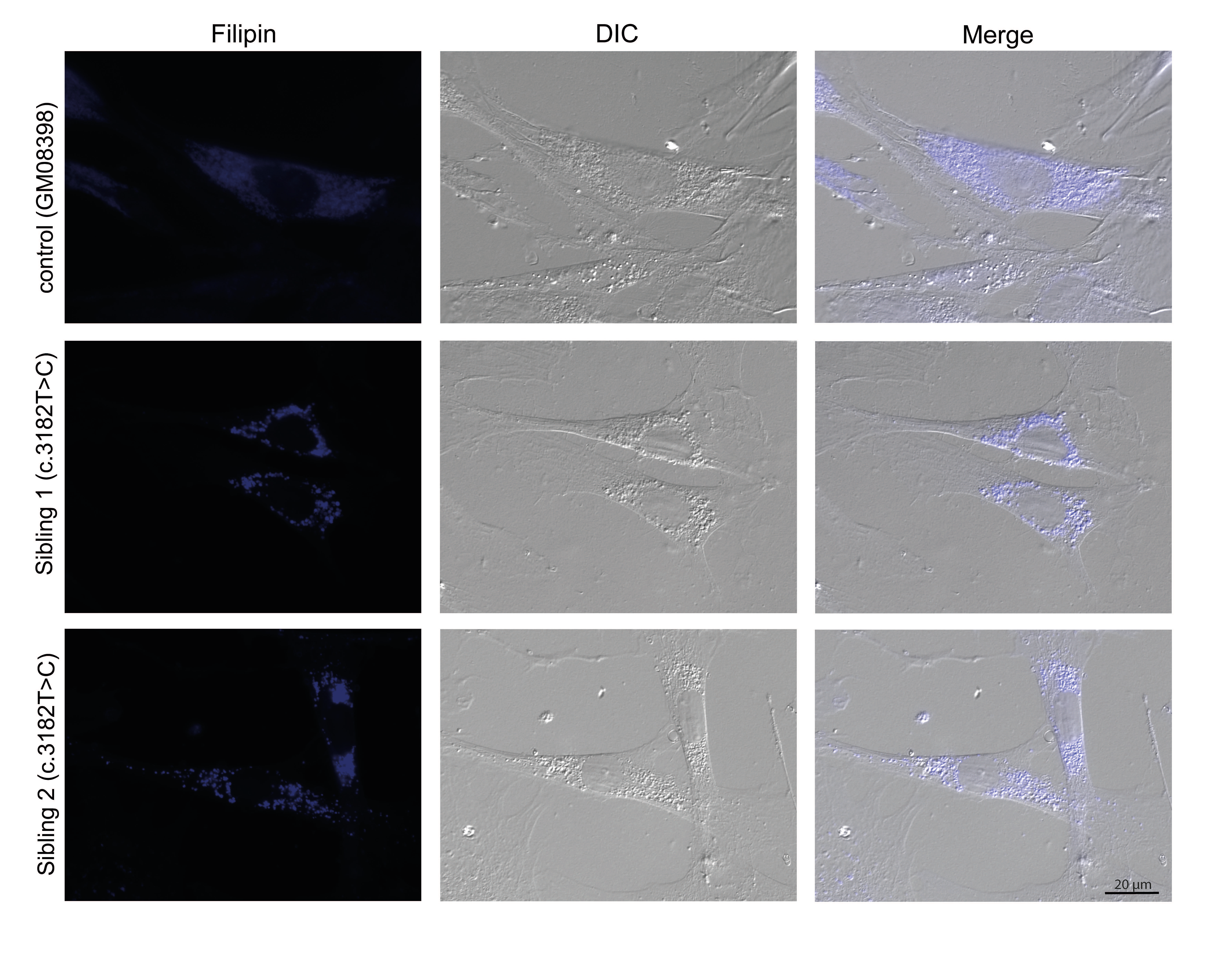


**Figure S5:** Verification of cholesterol deposits in patient-specific NPC1-deficient fibroblasts by filipin staining (blue). Fibroblasts of both patients showed a distinct accumulation of cholesterol, compared to a more homogeneous distribution in the control fibroblasts (GM08398). For better visualization of cell borders and localization of intracellular cholesterol, differential interference contrast (DIC) images were also captured. Scale bar: 20 µm.


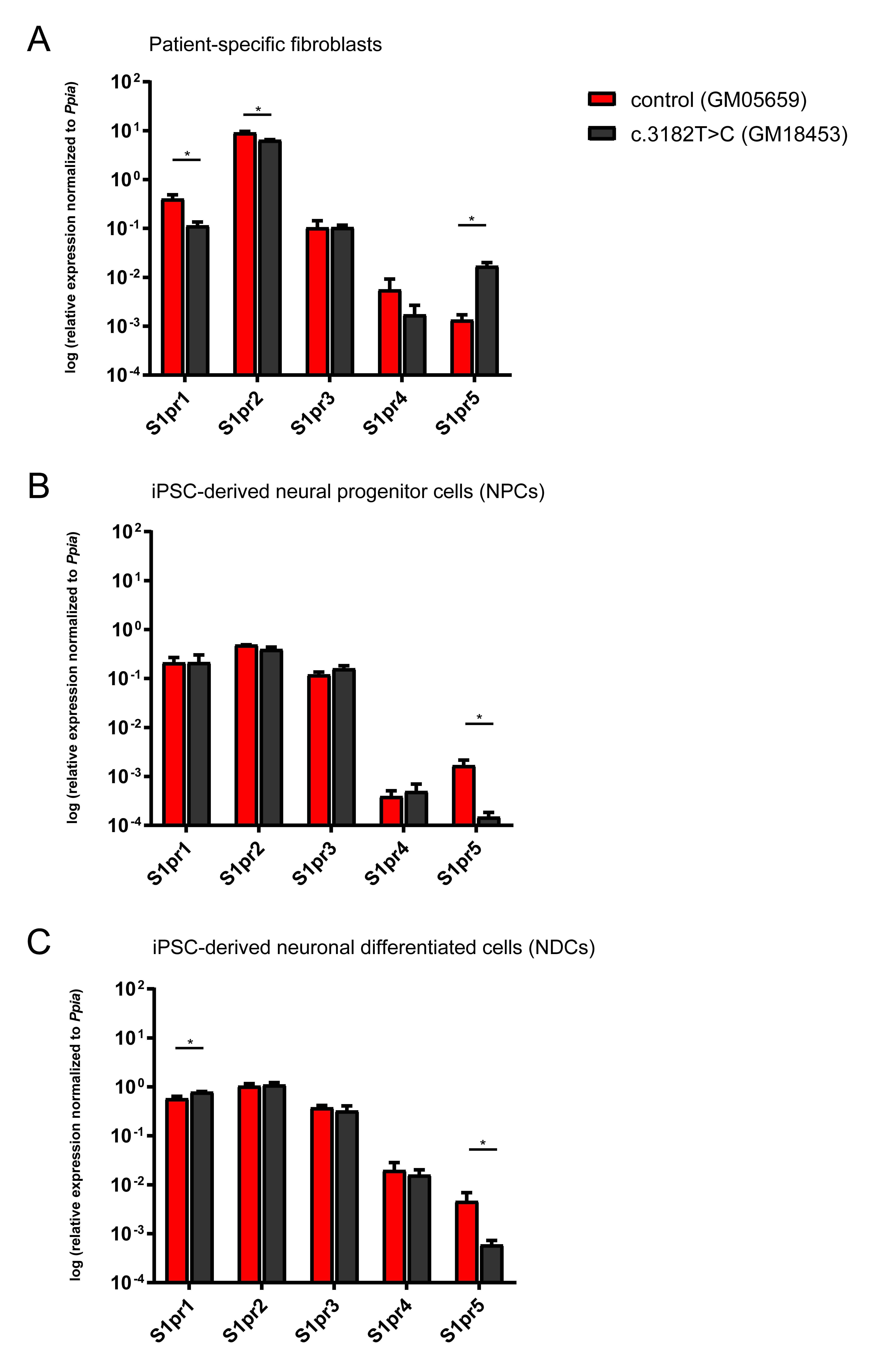


**Figure S6:** To investigate how far *S1pr1-5* expression is impaired in different human cell types (fibroblasts, NPCs, NDCs), qRT-PCR analyses were performed. NPC1 patient-specific fibroblasts (GM18453, c.3182T>C, Coriell Institute for Medical Research, **A**) showed reduced *S1pr1* and *S1pr2* mRNA expression and increased *S1pr5* mRNA expression compared to fibroblasts of an apparently healthy individual (GM05659, Coriell Institute for Medical Research). After reprogramming of NPC1 patient-specific fibroblasts, neural differentiation of iPSCs was induced, resulting in qRT-PCR analysis of NPCs (**B**) and NDCs (**C**). In contrast to fibroblasts, NPCs and NDCs showed a significant decrease of *S1pr5* expression. Likewise, in contrast to fibroblasts, NDCs showed a small significant increase in *S1pr1* expression. *S1pr5* expression presented the most distinct changes in the three different cell types, implicating *S1pr5* as an issue in NPC1 disease. Data are represented as mean ± SEM and are normalized to *Ppia* with n=4, that represents the data of 4 successive passages. NPCs: neural progenitor cells, NDCs: neuronal differentiated cells

**Table S1:** Mouse and human Taqman gene expression assays (FAM/MGB-coupled) used for qRT-PCR analysis of different brain regions, mouse primary cells and patient-specific fibroblasts (**A**). Mouse oligonucleotides used for cloning of GFP-coupled *S1pr1-5* constructs (**B**). Used restriction sites are labeled in red.


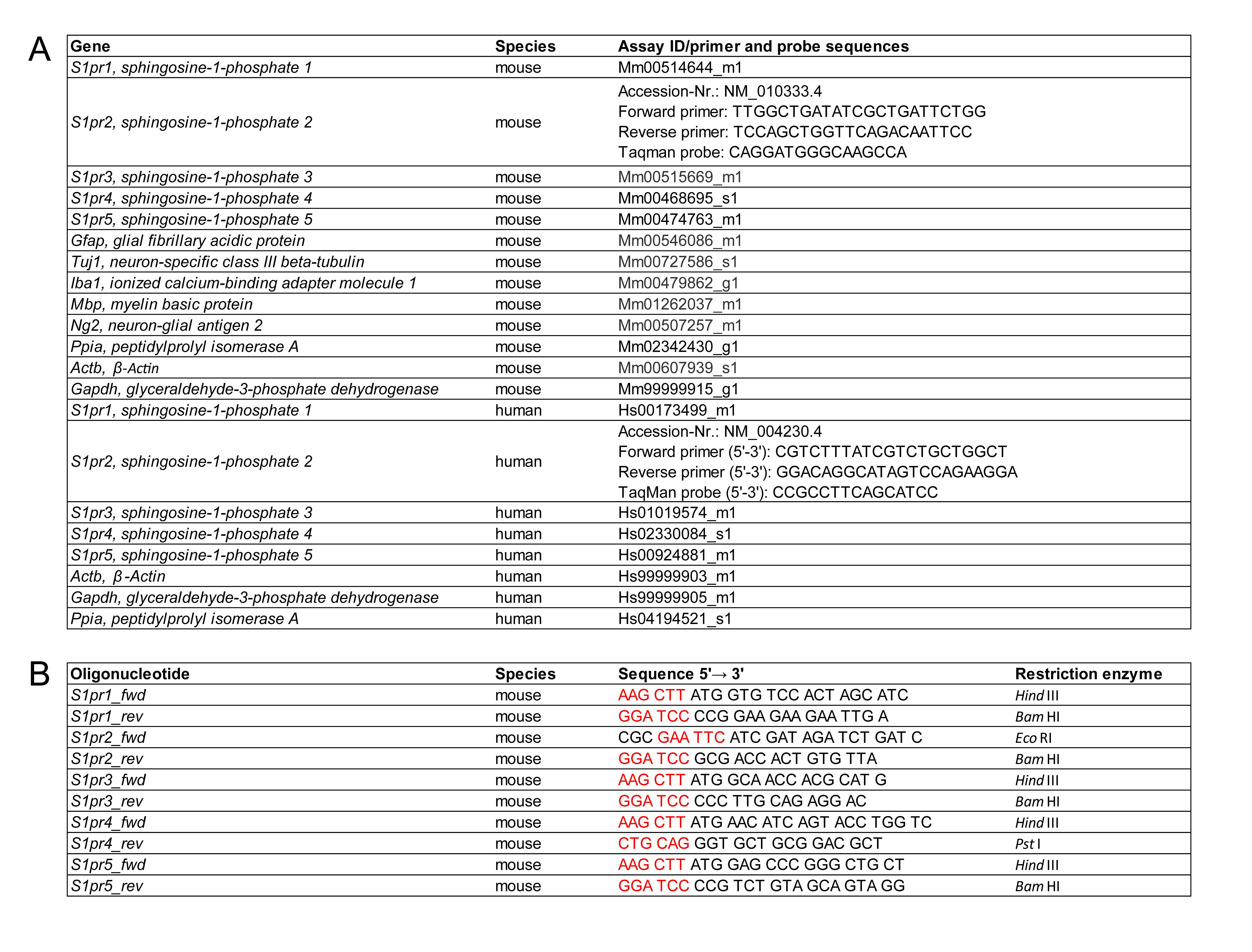


**Table S2:** Raw data of relative *S1pr1-5* mRNA expression from all brain regions of sham-treated and treated *Npc1^+/+^* and *Npc1^-/-^* mice (**A**). Data are normalized to *Ppia* and are represented as mean ± SEM; n=3. Statistical analysis was performed using two-tailed nonparametric Mann-Whitney-U-Test (**B**). p ≤ 0.05, labeled in red, was considered significant (*p ≤ 0.05).
